# Dynamics of the *Synechococcus elongatus* cytoskeletal GTPase FtsZ yields mechanistic and evolutionary insight into cyanobacterial and chloroplast FtsZs

**DOI:** 10.1016/j.jbc.2023.102917

**Published:** 2023-01-16

**Authors:** Katie J. Porter, Lingyan Cao, Katherine W. Osteryoung

**Affiliations:** Department of Plant Biology, Michigan State University, East Lansing, Michigan, USA

**Keywords:** *Synechococcus elongatus*, cyanobacteria, *Arabidopsis thaliana*, chloroplast, GTPase, FtsZ dynamics, chloroplast division, organelle division, endosymbiosis, AtFtsZ, *Arabidopsis thaliana* FtsZ, AtFtsZ1-mV, AtFtsZ1-mVenus, Cc, critical concentration, EcFtsZ, *Escherichia coli* FtsZ, FRAP, fluorescence recovery after photobleaching, FtsZ, filamenting temperature-sensitive Z, GsFtsZ, *Galdieria sulphuraria* FtsZ, LS, light scattering, LSB, low salt buffer, mVenus, mV, SeFtsZ, *Synechococcus elongatus* FtsZ, SeFtsZ-mC, SeFtsZ-mCerulean, SyFtsZ, *Synechocystis* FtsZ, TEM, transmission electron microscopy

## Abstract

The division of cyanobacteria and their chloroplast descendants is orchestrated by filamenting temperature-sensitive Z (FtsZ), a cytoskeletal GTPase that polymerizes into protofilaments that form a “Z ring” at the division site. The Z ring has both a scaffolding function for division-complex assembly and a GTPase-dependent contractile function that drives cell or organelle constriction. A single FtsZ performs these functions in bacteria, whereas in chloroplasts, they are performed by two copolymerizing FtsZs, called AtFtsZ2 and AtFtsZ1 in *Arabidopsis thaliana*, which promote protofilament stability and dynamics, respectively. To probe the differences between cyanobacterial and chloroplast FtsZs, we used light scattering to characterize the *in vitro* protofilament dynamics of FtsZ from the cyanobacterium *Synechococcus elongatus* PCC 7942 (SeFtsZ) and investigate how coassembly of AtFtsZ2 or AtFtsZ1 with SeFtsZ influences overall dynamics. SeFtsZ protofilaments assembled rapidly and began disassembling before GTP depletion, whereas AtFtsZ2 protofilaments were far more stable, persisting beyond GTP depletion. Coassembled SeFtsZ–AtFtsZ2 protofilaments began disassembling before GTP depletion, similar to SeFtsZ. In contrast, AtFtsZ1 did not alter disassembly onset when coassembled with SeFtsZ, but fluorescence recovery after photobleaching analysis showed it increased the turnover of SeFtsZ subunits from SeFtsZ–AtFtsZ1 protofilaments, mirroring its effect upon coassembly with AtFtsZ2. Comparisons of our findings with previous work revealed consistent differences between cyanobacterial and chloroplast FtsZ dynamics and suggest that the scaffolding and dynamics-promoting functions were partially separated during evolution of two chloroplast FtsZs from their cyanobacterial predecessor. They also suggest that chloroplasts may have evolved a mechanism distinct from that in cyanobacteria for promoting FtsZ protofilament dynamics.

Cyanobacteria are photosynthetic prokaryotes essential for life on earth. They possess a natural ability to harvest CO_2_ and introduce oxygen into the atmosphere and have many potential applications in bioremediation and the production of food, fuel, and other high-value compounds (1, 2, 3, 4). Cyanobacteria are also the endosymbiotic ancestors of eukaryotic chloroplasts (5). Therefore, studies of cyanobacteria inform studies of chloroplasts and vice versa, and probing the inner workings of both is important for understanding the biology, evolution, and ecology of photosynthetic organisms.

Like other bacteria, cyanobacteria as well as their chloroplast descendants proliferate by division (6, 7, 8). The central driver of division in most prokaryotes and chloroplasts is the tubulin-like cytoskeletal protein filamenting temperature-sensitive Z (FtsZ). FtsZ proteins form a dynamic “Z ring” at the site of division (9, 10, 11, 12). The Z ring acts as a scaffold for the recruitment additional proteins to the division site and is essential for constriction of the cell or organelle (8, 13, 14, 15, 16, 17).

FtsZ is a self-assembling GTPase that polymerizes into single-stranded polymers called protofilaments (18, 19, 20). FtsZ subunits assemble head to tail when one FtsZ binds GTP and another associates longitudinally, resulting in protofilament assembly. Because subunit association completes the active site for GTP hydrolysis, GTPase activity requires oligomerization (20, 21, 22). Hydrolysis within protofilaments reduces the affinity between GDP-bound subunits, leading to subunit disassociation from protofilament ends (23, 24). GTPase-dependent subunit exchange (turnover) results in treadmilling of *Escherichia coli* FtsZ (EcFtsZ) protofilaments, where assembly occurs preferentially at one end of the protofilament and disassembly occurs preferentially from the other end (14, 25, 26, 27). It is not yet known whether cyanobacterial or chloroplast FtsZ protofilaments treadmill, but GTPase-dependent protofilament turnover drives the contractile activity of both bacterial and chloroplast Z rings (28, 29, 30, 31).

Most of our understanding of FtsZ biochemistry and function comes from analysis of EcFtsZ (16). Despite the environmental and evolutionary importance of cyanobacteria, the FtsZs from only two cyanobacterial species have been biochemically characterized to date. One is from the spherical-shaped-cyanobacterium *Synechocystis* sp. PCC 6803 (32), a major model system for the study of photosynthesis (33). The other is from the filamentous cyanobacterium *Anabaena* sp. PCC 7120 (34), an important model for the study of nitrogen fixation (35, 36). Although *Synechococcus elongatus* PCC 7942 is the most extensively used model system for investigating the regulation of cyanobacterial cell division (6, 10, 37, 38, 39, 40), partly because it is rod shaped like *E. coli*, FtsZ has yet to be biochemically characterized in this important species. In addition, only a few studies have examined the biochemistry of the chloroplast FtsZs, in part because, unlike bacteria that have only a single FtsZ, most chloroplasts possess two nonredundant FtsZs that coassemble (41, 42, 43, 44, 45, 46, 47), adding complexity to their functional analysis.

Here, we carried out the first study of the *in vitro* assembly properties and dynamic behavior of *S. elongatus* PCC 7942 FtsZ (SeFtsZ). Because the chloroplast FtsZ proteins arose from the FtsZ in their cyanobacterial ancestor (41, 48, 49), we also investigated how coassembly of SeFtsZ with each of the FtsZs from *Arabidopsis thaliana* influences overall protofilament dynamics. Our results reveal significant differences in cyanobacterial and chloroplast FtsZ dynamics and yield surprising insight into changes in the mechanistic control of dynamics that may have accompanied the evolution of chloroplasts from cyanobacteria.

## Results

### Purified *S. elongatus* FtsZ hydrolyzes GTP and undergoes GTP-dependent assembly

**Figure 1:**
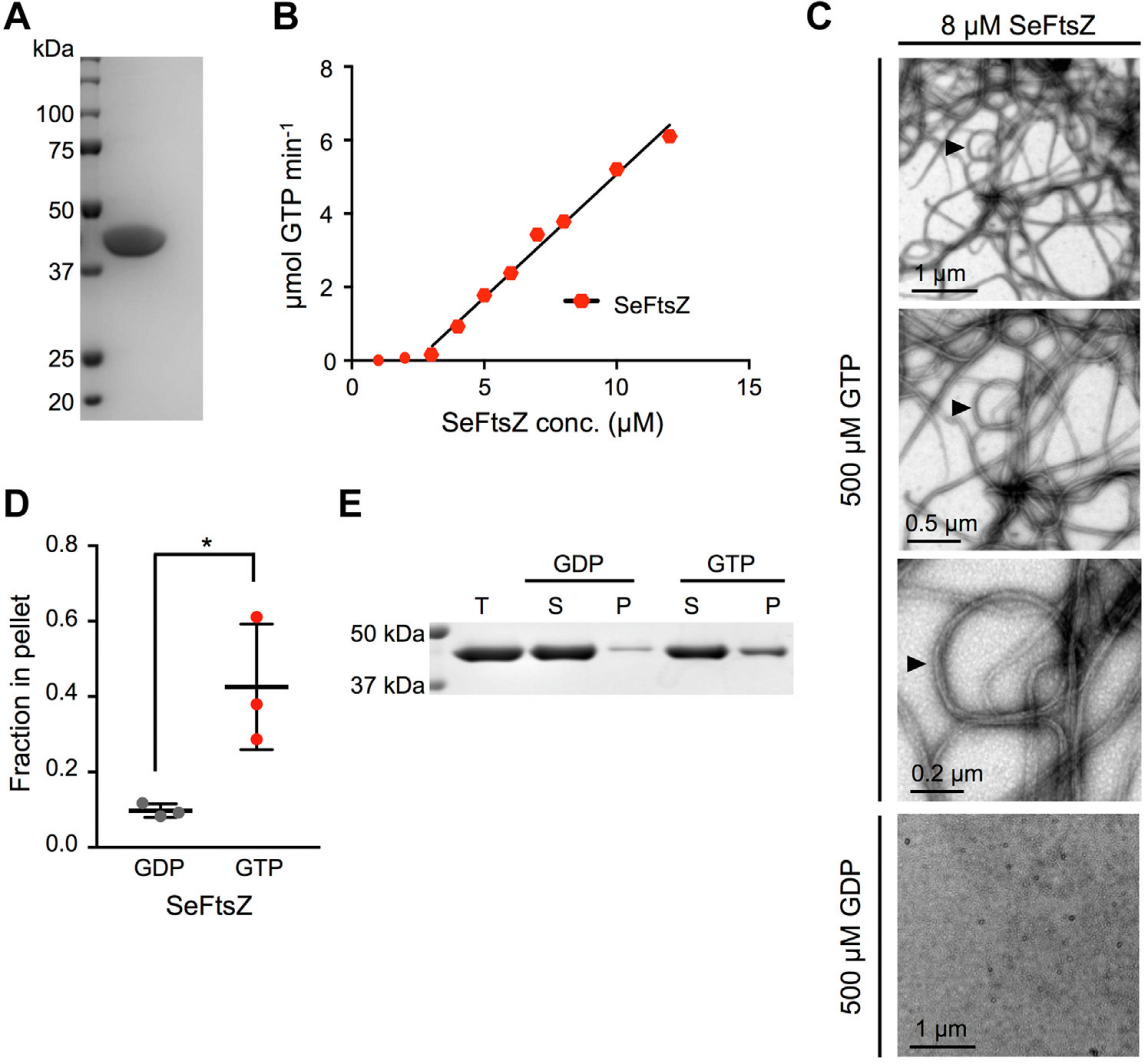
**SeFtsZ hydrolyzes GTP and undergoes GTP-dependent assembly.***A,* SDS-PAGE gel of purified SeFtsZ stained with Coomassie. Markers (kilodalton) are shown on the *left*. *B,* GTPase activity of SeFtsZ. GTPase hydrolysis rates (μM GTP min^−1^) were assayed in 500 μM GTP at pH 7.5 and 25 °C at protein concentrations ranging from 2 to 14 μM. A representative assay is shown. The GTPase activity (μM GTP min^−1^ FtsZ^−1^) is the slope of the regression line above the critical concentration, which is the x-intercept. The regression line was fitted to the hexagonal symbols. *C,* negative-stain transmission electron microscopy of 8 μM SeFtsZ incubated for 5 min at room temperature with either 500 μM GTP (*top three panels*) or GDP (*bottom panel*). For assembly in GTP, the *middle* and *lower panels* are higher magnifications of the *top panel*, which was taken from the *upper left quadrant* of the wider field imaged in the *top left panel* of Figure 2*E*. Scale bars are as indicated. *D* and *E,* sedimentation assays. Reactions containing 6 μM SeFtsZ were incubated for 30 min at room temperature after addition of either 500 μM GTP or GDP and then centrifuged at 80,000*g* for 30 min at 4 °C. *D,* fraction of SeFtsZ protein in the pellet (n = 3). The difference between the fraction of SeFtsZ in the pellet in GDP *versus* GTP was statistically significant at *p* = 0.027 (∗). *E,* representative Coomassie-stained SDS-PAGE gel of SeFtsZ protein in the total (T), supernatant (S), or pellet (P). Markers (kilodalton) are shown on the *left*. SeFtsZ, *Synechococcus elongatus* FtsZ.

We began our characterization of SeFtsZ by expressing and purifying soluble His-tagged SeFtsZ. Nickel–nitrilotriacetic acid purification produced a single band on SDS-PAGE gels (Fig. 1*A*). To determine the GTPase activity of SeFtsZ, GTP hydrolysis rates were measured at protein concentrations ranging from 2 to 14 μM and plotted, and the activity was taken as the slope of the resulting regression line of activities above 0 (Fig. 1*B*). The GTPase activity of SeFtsZ was 0.59 ± 0.13 GTP min^−1^ FtsZ^−1^ (Table 1). Because GTP hydrolysis by FtsZ proteins requires oligomerization, their GTPase activities exhibit a critical concentration (Cc), that is, a minimal FtsZ concentration above which polymerization occurs and hence GTPase activity is detectable (50). The Cc for SeFtsZ activity, estimated from the extrapolated X-intercept of the GTPase plot (51), was 2.12 ± 0.35 μM (Fig. 1*B* and Table 1). This is close to the Cc of *Synechocystis* sp. PCC 6803 FtsZ (32) but higher than those of the *A. thaliana* chloroplast proteins (47), indicating the cyanobacterial FtsZ subunits interact with lower affinity (45).

**Table 1:** GTPase activities and critical concentrations for SeFtsZ and SeFtsZ in mixture with AtFtsZ2 or AtFtsZ1 at various ratios

Protein components (ratios in GTPase assays)	Concentrations in coassembly assays (μM:μM)	GTPase activity (GTP min^−1^ FtsZ^−1^)	Cc (μM)
SeFtsZ	N/A	0.59 ± 0.13 (n = 7)	2.12 ± 0.35
AtFtsZ2	N/A	0.21 ± 0.02 (n = 2)	0.70 ± 0.24
SeFtsZ:AtFtsZ2 (0.1:1)	0.08:8	0.29 ± 0.01 (n = 2)	1.55 ± 0.15
SeFtsZ:AtFtsZ2 (0.2:1)	1.6:8	0.35 ± 0.06 (n = 2)	1.42 ± 0.85
SeFtsZ:AtFtsZ2 (0.5:1)	4:8, 2.66:5.33	0.38 ± 0.09 (n = 2)	1.67 ± 1.07
SeFtsZ:AtFtsZ2 (1:0.1)	8:0.8	0.86 ± 0.008 (n = 2)	1.17 ± 0.09
SeFtsZ:AtFtsZ2 (1:0.2)	8:1.6	0.92 ± 0.006 (n = 2)	1.46 ± 0.15
SeFtsZ:AtFtsZ2 (1:0.5)	8:4	0.75 ± 0.02 (n = 2)	1.50 ± 0.31
SeFtsZ:AtFtsZ2 (1:1)	4:4	0.62 ± 0.05 (n = 3)	2.68 ± 0.07
AtFtsZ1	N/A	0.40 ± 0.05 (n = 3)	0.11 ± 0.29
SeFtsZ:AtFtsZ1 (1:0.1)	8:0.8	0.34 ± 0.06 (n = 2)	1.29 ± 0.46
SeFtsZ:AtFtsZ1 (1:0.2)	8:1.6	0.46 ± 0.04 (n = 2)	1.30 ± 0.44
SeFtsZ:AtFtsZ1 (1:0.5)	8:4	0.54 ± 0.08 (n = 3)	1.85 ± 0.26

Abbreviation: N/A, not available.

GTPase activities were calculated based on total FtsZ concentration. Values represent the average of the indicated number of assays (n) ± SD. All reactions were performed with 500 μM GTP. Actual protein concentrations used in coassembly experiments are also shown.

Negative-stain transmission electron microscopy (TEM) was used to visualize SeFtsZ assembly. Assembly was initiated by addition of GTP. After 300 s, 8 μM SeFtsZ in 500 μM GTP assembled into straight or curved bundles of protofilaments with an average thickness of 29.17 ± 1.7 nm (Fig. 1*C*, *top three panels*). No protofilaments were observed in 500 μM GDP (Fig. 1*C*, *bottom panel*), confirming that assembly was GTP dependent. In keeping with TEM observations, sedimentation assays demonstrated a significant increase of SeFtsZ in the pellet fraction following incubation in 500 μM GTP compared with 500 μM GDP (Fig. 1, *D* and *E*). Curved protofilament bundles sometimes formed toroids with an average internal diameter of 341.72 ± 13.09 nm (Fig. 1*C*).

### SeFtsZ disassembles as GTP is consumed

*In vitro* light scattering (LS) assays have shown that EcFtsZ typically undergoes rapid assembly upon addition of GTP, and rapid disassembly as GTP hydrolysis reduces protofilament stability (23, 52, 53). In contrast, we found that *A. thaliana* FtsZ2 (AtFtsZ2), one of the two FtsZs in *A. thaliana* chloroplasts (41, 42), disassembled very slowly even after GTP was fully consumed, indicating AtFtsZ2 protofilaments are far more stable (47). Given the evolutionary relationship between cyanobacterial and chloroplast FtsZs (41, 48, 49), we were interested in the dynamic behavior of SeFtsZ and used LS assays (47, 52) to investigate its assembly and disassembly dynamics.

**Figure 2:**
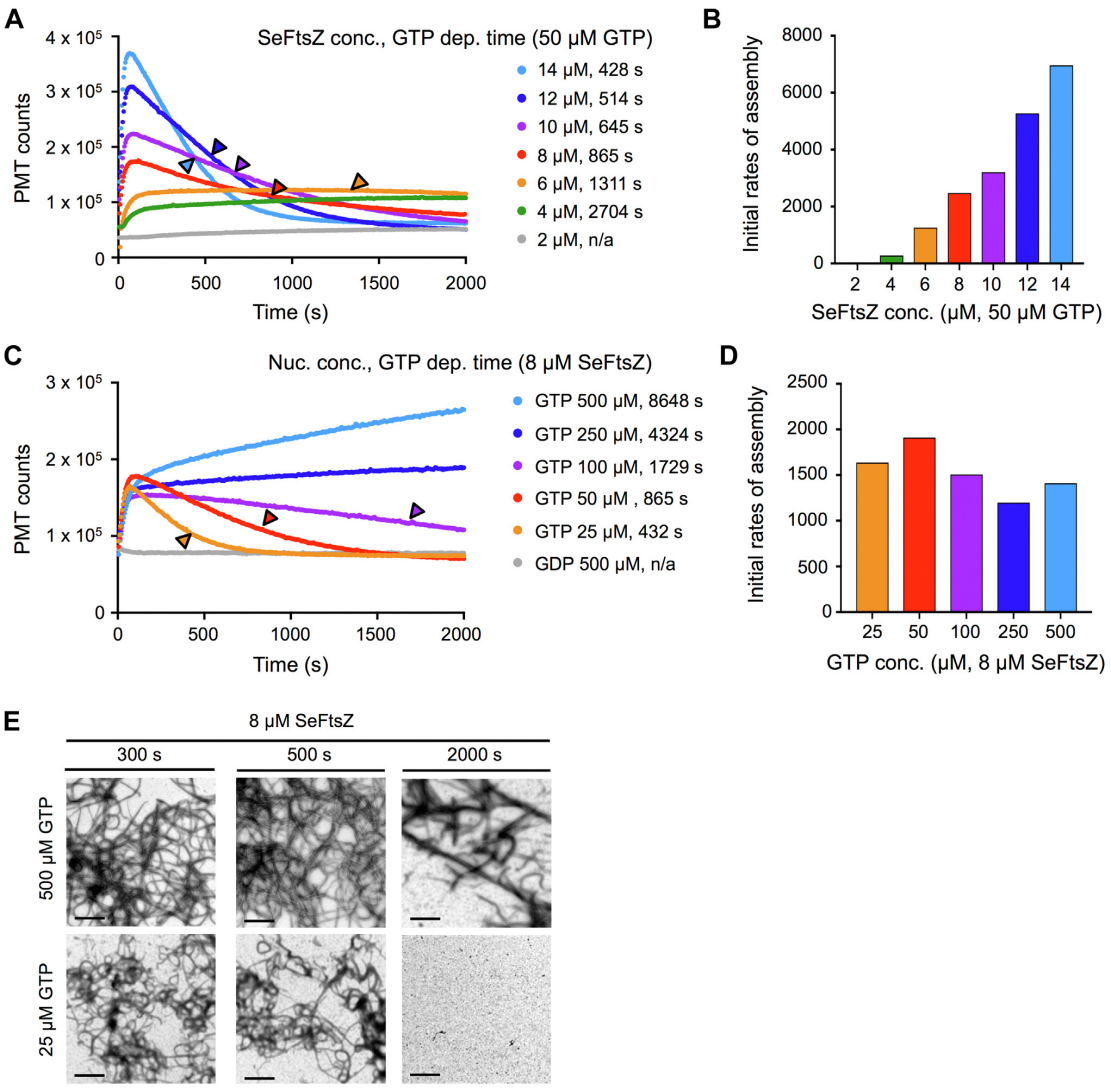
**SeFtsZ assembles rapidly and disassembles as GTP is hydrolyzed.** Assembly reactions were performed at room temperature, initiated by addition of nucleotide, and repeated at least twice with similar results. *A,* assembly of SeFtsZ monitored by light scattering (LS) at the indicated protein concentrations after addition of 50 μM GTP. Predicted times of GTP depletion (GTP dep. time) are shown next to protein concentrations and indicated by *arrowheads*. *B,* initial rates of assembly for the LS traces in *A*. *C,* LS assays of 8 μM SeFtsZ after addition of GTP or GDP at the indicated concentrations. Predicted GTP depletion times are shown next to nucleotide concentrations and indicated by *arrowheads* where they fall within the 2000 s assay period. *D,* initial rates of assembly for the LS traces in *C*. *E,* negative-stain transmission electron microscopy of 8 μM SeFtsZ incubated for 300 s (*left column*), 500 s (*middle column*), or 2000 s (*right column*) after addition of 500 μM GTP (*top row*) or 25 μM GTP (*bottom row*). The scale bar represents 1 μm. SeFtsZ, *Synechococcus elongatus* FtsZ.

LS assays were initiated by addition of GTP and carried out over 2000 s. To facilitate detection of disassembly, we chose to identify an initiating GTP concentration that would ensure complete GTP hydrolysis during this period. We therefore calculated predicted GTP depletion times at different SeFtsZ and GTP concentrations based on the GTPase activity measured at 500 μM GTP (Fig. 1*B* and Table 1) and found that 50 μM GTP predicted depletion times between 428 s at 14 μM SeFtsZ and 2704 s at 4 μM SeFtsZ. GTPase assays confirmed that SeFtsZ activities were equivalent at 50 and 500 μM GTP (Fig. S1). LS assays were therefore initiated by addition of 50 μM GTP. During the 2000 s of monitoring, an initial increase in LS, indicative of assembly, was observed at 4 μM SeFtsZ and above (Fig. 2*A*). Both the amplitude of assembly and initial rate of assembly increased as the concentration of SeFtsZ increased (Fig. 2, *A* and *B*). Little to no assembly occurred at 2 μM SeFtsZ (Fig. 2*A*, *gray trace*), consistent with the Cc of 2.12 μM (Table 1). No disassembly was observed at 4 μM SeFtsZ, in agreement with predicted GTP depletion at 2704 s (Fig. 2*A*; *green trace*). At all higher SeFtsZ concentrations, disassembly did occur as indicated by the decrease in LS (Fig. 2*A*). Disassembly began before predicted GTP depletion (Fig. 2*A*, *arrowheads*) and was more rapid at higher concentrations, as the greater protein present in the reaction consumed GTP sooner.

To further explore the relationship between disassembly and nucleotide consumption, we performed LS assays on a fixed concentration of SeFtsZ (8 μM) and varied GTP concentration between 25 and 500 μM. In a control assay, no assembly occurred in 500 μM GDP (Fig. 2*C*, *gray trace*). In all reactions with GTP, SeFtsZ assembled to similar amplitudes and at similar initial rates (Fig. 2, *C* and *D*). Combined with the data in Figure 2, *A* and *B*, these results indicate that assembly under these conditions is limited by SeFtsZ concentration, not GTP. Disassembly was observed at 25, 50, and 100 μM GTP, where GTP depletion was predicted within 2000 s, but not at 250 or 500 μM GTP, where depletion was much later (Fig. 2*C*). TEM confirmed the LS results; protofilaments were visible at 300, 500, and 2000 s in 500 μM GTP but only at 300 and 500 s in 25 μM GTP (Fig. 2*E*), consistent with the LS signals at these time points (Fig. 2*C*, *blue and orange traces*). Taken together, our LS experiments show that SeFtsZ assembles rapidly and disassembles as GTP is hydrolyzed, revealing its assembly dynamics are much more similar to those of EcFtsZ than the more stable chloroplast AtFtsZ2.

### SeFtsZ reduces AtFtsZ2 stability

The single FtsZ present in bacteria must impart enough stability for protofilaments to support their scaffolding function within the Z ring while also allowing sufficient GTPase-dependent subunit turnover to drive protofilament and Z-ring dynamics (16). By contrast, we have shown that the *A. thaliana* chloroplast proteins AtFtsZ2 and AtFtsZ1, which coassemble, play distinct roles in promoting protofilament stability and dynamics, respectively (30, 47). This suggests partial separation of these complementary functions during the evolution of chloroplasts from cyanobacteria. To further explore the differences between cyanobacterial and chloroplast FtsZs, we used LS assays to investigate how coassembly of SeFtsZ with the AtFtsZ proteins would affect protofilament dynamics.

**Figure 3:**
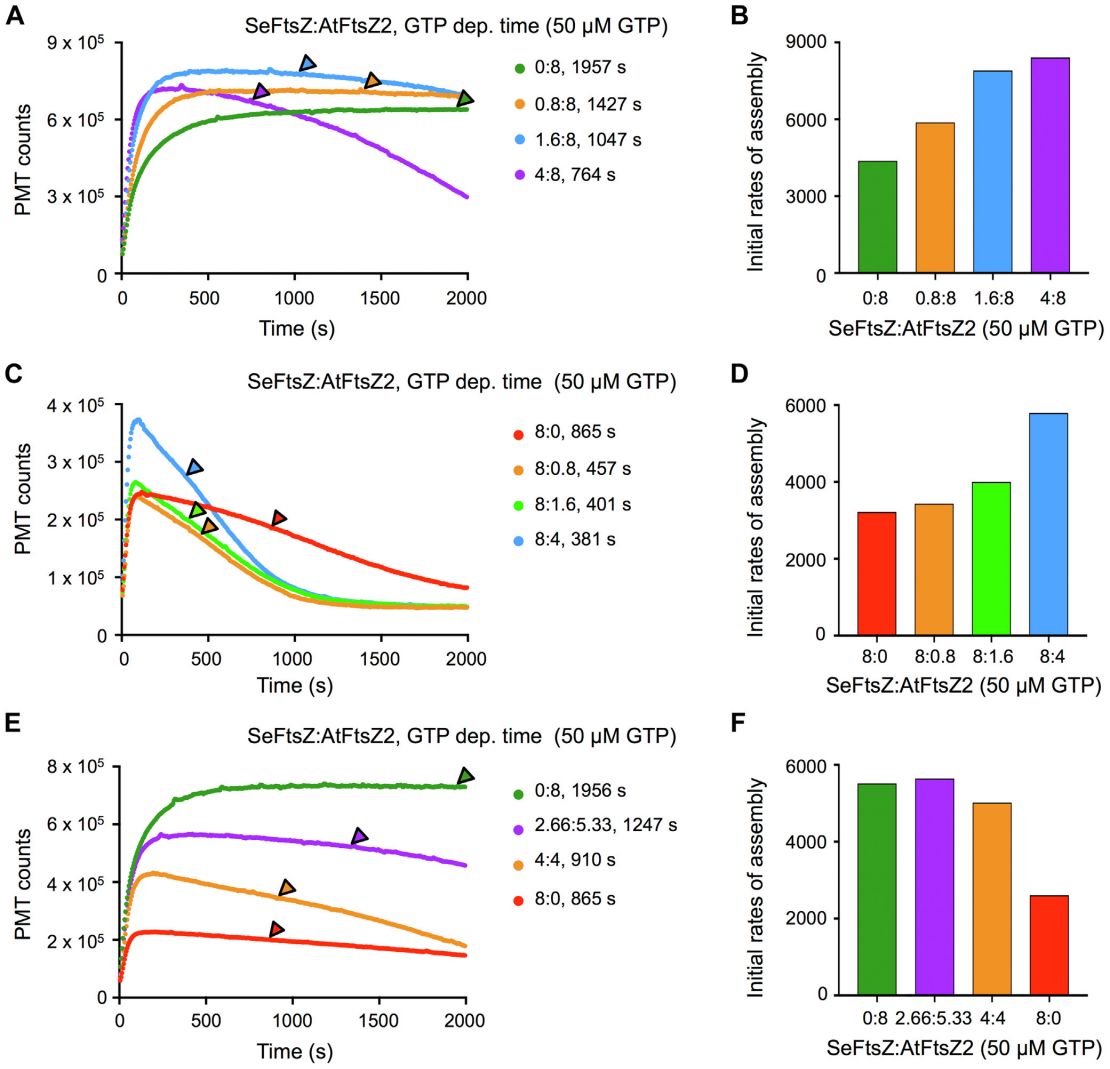
**Coassembly of SeFtsZ with AtFtsZ2 reduces AtFtsZ2 stability.** Assembly reactions were performed at room temperature, initiated by addition of nucleotide and repeated at least twice with similar results. *A, C,* and *E,* assembly of SeFtsZ and AtFtsZ2 monitored by light scattering (LS) at the indicated SeFtsZ:AtFtsZ2 concentration ratios (μM:μM) after addition of 50 μM GTP. Predicted times of GTP depletion (GTP dep. time) are shown next to protein concentrations and indicated by *arrowheads*. *B, D*, and *F,* initial rates of assembly for the LS traces in *A, C*, and *E*, respectively. AtFtsZ, *Arabidopsis thaliana* FtsZ; SeFtsZ, *Synechococcus elongatus* FtsZ.

Because of the very different stabilities of SeFtsZ and AtFtsZ2, we first asked how their coassembly might affect overall protofilament stability. In the first set of experiments, 8 μM AtFtsZ2 was mixed with 0.8, 1.6, or 4 μM SeFtsZ (SeFtsZ:AtFtsZ2 ratios of 0.1:1, 0.2:1, and 0.5:1, respectively). GTPase activities at these ratios were measured (Fig. S2 and Table 1), and the time of GTP depletion at each ratio was calculated. LS assays were initiated by addition of 50 μM GTP. In a control assay with AtFtsZ2 alone, no disassembly was observed during monitoring despite GTP depletion (Fig. 3*A*, *green trace*), demonstrating the exceptional stability of AtFtsZ2 shown previously (47). The addition of SeFtsZ resulted in increases in the amplitudes and initial rates of assembly (Fig. 3, *A* and *B*). As evidence that these changes reflected coassembly and not separate assembly, both the amplitudes of assembly and the GTPase activities in the mixtures containing 0.8 and 1.6 μM SeFtsZ were higher than those of AtFtsZ2 alone (Fig. 3*A* and Table 1) even though these concentrations are below the Cc of 2.12 μM where SeFtsZ cannot assemble and therefore cannot hydrolyze GTP on its own (Figs. 1*B* and 2A, *gray trace*). Some disassembly occurred in all the mixtures and began before GTP was depleted (Fig. 3*A*), indicating that coassembled protofilaments are less stable than AtFtsZ2 protofilaments.

We then mixed 8 μM SeFtsZ with 0.8, 1.6, or 4 μM AtFtsZ2. At the two lower AtFtsZ2 concentrations, there was little change in the amplitude or initial rate of assembly compared with those of SeFtsZ alone (Fig. 3, *C* and *D*). Substantial increases in both were observed at 4 μM AtFtsZ2 (Fig. 3, *C* and *D*). In all three mixtures, the GTPase activities were higher than that of SeFtsZ (Table 1), resulting in earlier predicted times of GTP depletion (Fig. 3*C, arrowheads*). Similar to the behavior of SeFtsZ alone and the mixtures with 8 μM AtFtsZ2, disassembly in the mixtures with 8 μM SeFtsZ began before GTP depletion but occurred more rapidly and was more complete (Fig. 3, *A* and *C*).

In the aforementioned coassembly experiments, the predicted GTP depletion times at different ratios were influenced by differences in total protein concentration as well as GTPase activity. To better assess the relationship between GTP consumption and protofilament stability in the mixtures, we carried out coassembly assays at a fixed total protein concentration of 8 μM. Under these conditions, differences in GTP depletion times are only because of differences in GTPase activities. The amplitude of assembly was highest for AtFtsZ2, decreased as the SeFtsZ:AtFtsZ2 ratio increased from 2.66:5.33 to 4:4 μM, and was lowest for SeFtsZ (Fig. 3*E*). Disassembly began before GTP depletion in both mixtures. Combined with the aforementioned data, these results demonstrate that the onset of disassembly before GTP depletion is not due simply to faster GTP consumption at higher protein concentrations but is rather a more direct effect of SeFtsZ on the stability of the coassembled SeFtsZ–AtFtsZ2 protofilaments. Together, our results show that SeFtsZ has the ability to reduce AtFtsZ2 stability.

### AtFtsZ1 restrains SeFtsZ assembly and increases SeFtsZ subunit turnover

**Figure 4:**
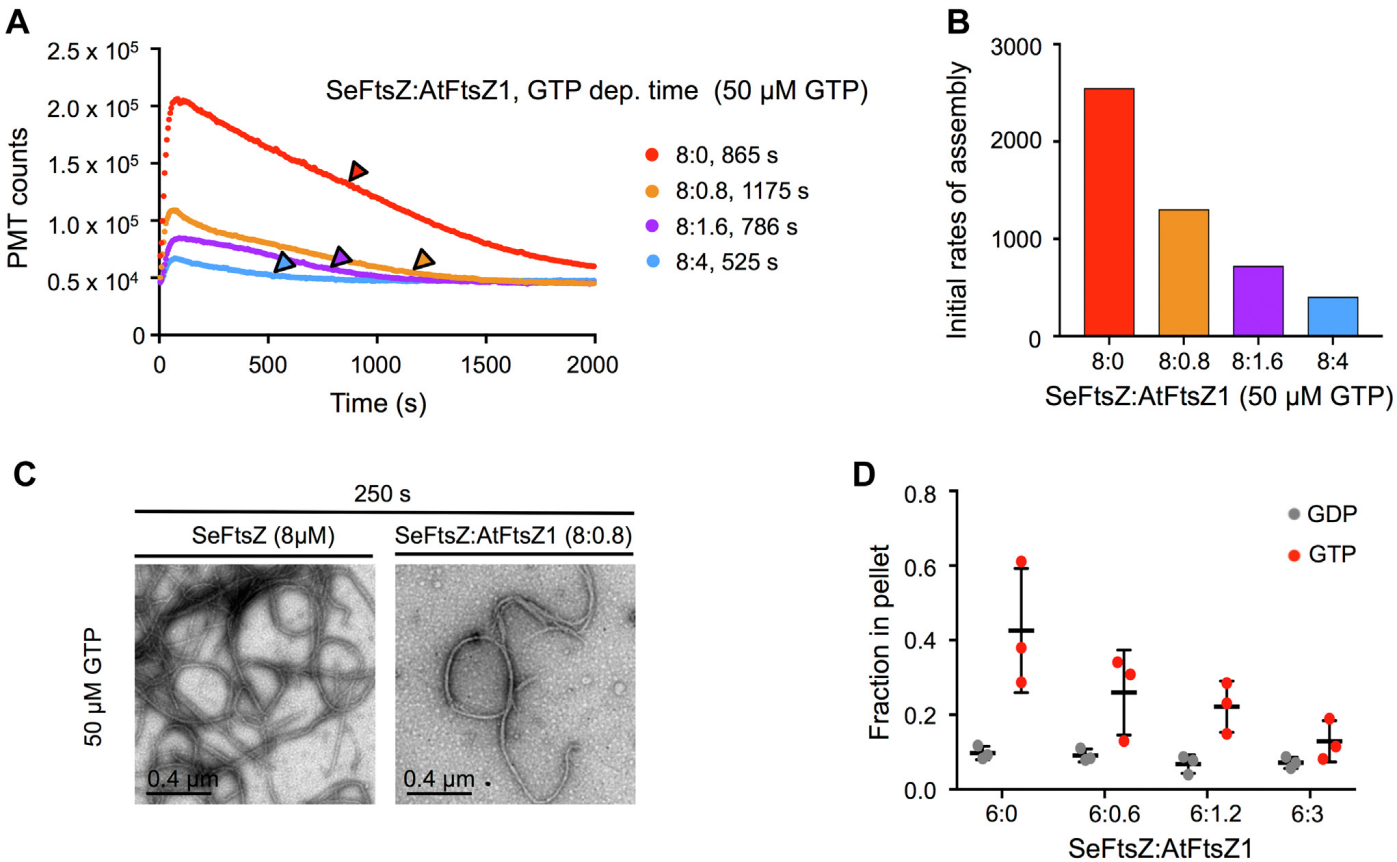
**AtFtsZ1 reduces assembly of SeFtsZ *in vitro.*** Assembly reactions were performed at room temperature, initiated by addition of nucleotide and repeated at least twice with similar results. *A,* assembly of SeFtsZ and AtFtsZ1 monitored by light scattering (LS) at the indicated SeFtsZ:AtFtsZ1 concentration ratios (μM:μM) after addition of 50 μM GTP. Predicted times of GTP depletion (GTP dep. time) are shown next to protein concentrations and indicated by *arrowheads*. *B,* initial rates of assembly for the LS traces in *A*. *C,* negative-stain transmission electron microscopy of 8 μM SeFtsZ (*left*) and 8 μM SeFtsZ with 0.8 μM AtFtsZ1 (*right*) incubated for 250 s after addition of 50 μM GTP. *D,* sedimentation assays. Reactions containing 6 μM SeFtsZ and AtFtsZ1 mixed at different ratios were initiated by addition of either 500 μM GTP or GDP and performed as described for Figure 1. Fractions of SeFtsZ protein in the pellet are shown (n = 3). The 6:0 μM SeFtsZ data are repeated from Figure 1*D*. Representative Coomassie-stained SDS-PAGE gels can be found in Fig. S3. AtFtsZ, *Arabidopsis thaliana* FtsZ; SeFtsZ, *Synechococcus elongatus* FtsZ.

In previous work, assembly of AtFtsZ1 by itself could not be detected *in vitro*, but its coassembly with AtFtsZ2 constrained the amplitude of assembly and enhanced the turnover of AtFtsZ2 subunits from AtFtsZ2–AtFtsZ1 protofilaments without increasing their GTPase activity (30, 47). To test whether these properties are unique to AtFtsZ2–AtFtsZ1 coassembly, we used LS assays to ask whether and how AtFtsZ1 would affect assembly of SeFtsZ. At the lowest SeFtsZ:AtFtsZ1 ratio (8:0.8 μM; 1:0.1), the amplitude and initial rate of assembly were substantially lower than for 8 μM SeFtsZ alone and decreased further as AtFtsZ1 was increased (Fig. 4, *A* and *B*). TEM confirmed these findings; after 250 s of assembly, protofilament morphologies were similar for SeFtsZ and the 1:0.1 mixture, but a reduction in protofilament abundance was evident in the latter (Fig. 4*C*). A decrease in SeFtsZ sedimentation was also observed with increasing AtFtsZ1 (Figs. 4*D* and S3). In all LS reactions disassembly commenced prior to GTP depletion (Fig. 4*A*). The GTPase activities at all ratios were lower than that of SeFtsZ (Table 1 and Fig. S2), indicating that the earlier predicted depletion times at the higher AtFtsZ1 ratios (Fig. 4*A*) were due to increased total protein concentration and not increased GTPase activity. Notably, the depletion times for SeFtsZ and 8:1.6 μM SeFtsZ:AtFtsZ1 were similar, indicating that the reduced amplitude of assembly in the mix was due to a constraint on the initial assembly rather than accelerated disassembly because of GTP hydrolysis. These results show that AtFtsZ1 is capable of coassembling with and restraining the assembly of SeFtsZ without increasing overall GTPase activity, similar to its effect on AtFtsZ2.

**Figure 5:**
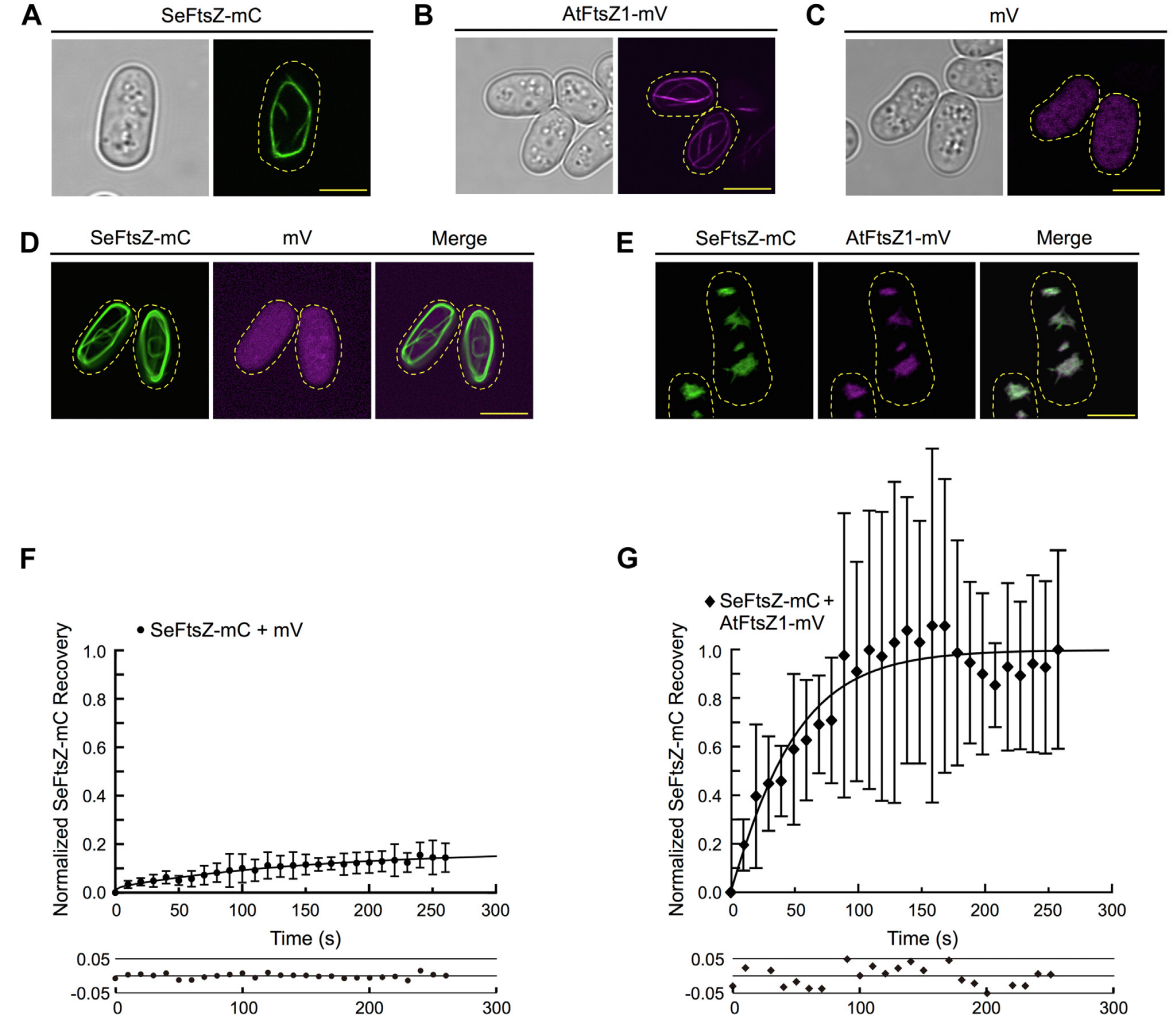
**AtFtsZ1 increases SeFtsZ subunit turnover.***A*–*E,* localization of SeFtsZ-mCerulean (SeFtsZ-mC, *green*), AtFtsZ1-mVenus (AtFtsZ1-mV, *magenta*), and mVenus (mV, *magenta*) in *Schizosaccharomyces pombe* strains expressing the indicated proteins. In fluorescence images, the *yellow dashed lines* show the outlines of the imaged cells. Scale bars represent 5 μm. *A*–C, cells expressing SeFtsZ-mC (*A*), AtFtsZ1-mV (*B*), or mV (*C*). *Left panels* show the same cells imaged using differential interference contrast. *D* and *E,* coexpression of SeFtsZ-mC (*D* and *E; left*) with either mV (*D*; *middle*) or AtFtsZ1-mV (*E*; *middle*) and the resulting merged images (*D* and *E; right*). *F* and *G,* FRAP analysis of SeFtsZ-mC in cells coexpressing SeFtsZ-mC with mV (*F*) or AtFtsZ1-mV (*G*). Recovery curves are normalized against background fluorescence at each time point. The *lower plots* show that all data points are within 0.05 normalized recovery units of the fitted curves (31, 65). Error bars represent SD. AtFtsZ, *Arabidopsis thaliana* FtsZ; FRAP, fluorescence recovery after photobleaching; SeFtsZ, *Synechococcus elongatus* FtsZ.

Fluorescence recovery after photobleaching (FRAP) experiments in the yeast *Schizosaccharomyces pombe* showed that AtFtsZ1 significantly enhances AtFtsZ2 turnover from AtFtsZ2–AtFtsZ1 protofilaments in living cells (30). We carried out comparable FRAP experiments to test whether AtFtsZ1 would similarly affect SeFtsZ. To this end, we first expressed and visualized the fluorescent fusion proteins SeFtsZ-mCerulean (SeFtsZ-mC) and AtFtsZ1-mVenus (AtFtsZ1-mV) in *S. pombe*. Each formed mostly long curved or ring-shaped cables when expressed separately (Fig. 5, *A* and *B*). Because we found previously that an unfused fluorophore reduced AtFtsZ turnover in FRAP assays (54), we also coexpressed SeFtsZ-mC with unfused mV for use as the minus-AtFtsZ1 FRAP control. SeFtsZ-mC localized to cables and mV in a diffuse pattern whether expressed singly or together (Fig. 5, *A*, *C* and *D*), indicating that the mV fluorophore itself did not impair SeFtsZ-mC assembly. In contrast, in cells coexpressing SeFtsZ-mC and AtFtsZ1-mV, the two proteins colocalized in patches, many of which appeared to contain very short filaments (Fig. 5*E*). The colocalization is consistent with coassembly.

We then performed FRAP assays on SeFtsZ-mC coexpressed with either mV or AtFtsZ1-mV. The recovery of SeFtsZ-mC fluorescence after photobleaching was much lower in the presence of mV (about 17%) than AtFtsZ1-mV (about 95%) (Figs. 5, *F* and *G* and S4). These results show that AtFtsZ1-mV strongly enhanced SeFtsZ-mC subunit turnover from the assembled structures. We suggest that the elevated turnover accounts for both the very short length of filaments in the coexpression strains (Fig. 5*E*) and the greatly reduced amplitudes and initial rates of assembly in the mixtures (Fig. 4, *A* and *B*). Together, our SeFtsZ–AtFtsZ1 coassembly experiments reveal that the ability of AtFtsZ1 to enhance the dynamics of protofilaments without stimulating their overall GTPase activity (Table 1) is not restricted to its interaction with AtFtsZ2 and is likely an intrinsic property of its coassembly behavior.

## Discussion

Our work brings to focus similarities and differences between the evolutionarily related cyanobacterial and chloroplast FtsZs. The GTPase activity of SeFtsZ was comparable to the values reported for *Synechocystis* sp. PCC 6803 FtsZ (SyFtsZ) and *Anabaena* sp. PCC 7120 FtsZ as well as AtFtsZ1, AtFtsZ2, and the two chloroplast FtsZs from the red alga *Galdieria sulphuraria*, GsFtsZA and GsFtsZB (32, 34, 45, 47, 55). The activities of these proteins range from about 0.4 to 1 GTP min^−1^ FtsZ^−1^, which is roughly an order of magnitude lower than those typically reported for EcFtsZ (24, 51, 56, 57, 58). Low GTPase activity may therefore be a conserved feature of cyanobacterial and chloroplast FtsZs. All these proteins also form protofilament bundles *in vitro* (32, 34, 45, 47, 55) except AtFtsZ1, which does not assemble under our assay conditions (47). SeFtsZ bundles sometimes formed toroids (Fig. 1*C*), as did SyFtsZ and *Anabaena* sp. PCC 7120 FtsZ (32, 34), all in dilute buffer. In contrast, EcFtsZ and *Mycobacterium tuberculosis* FtsZ required crowding agents to form toroids, which were proposed to represent energy-minimized condensates (59, 60). Although toroid formation *in vitro* has not been observed for the AtFtsZ or GsFtsZ proteins (45, 46, 47), it was for *Medicago truncatula* FtsZ1 (61). In addition, FtsZ “minirings,” possibly representing toroids, have been noted in a few *A. thaliana* mutants defective in chloroplast division (62, 63, 64). The *in vivo* relevance of bundle and toroid formation *in vitro*, however, remains elusive.

While cyanobacterial and chloroplast FtsZs exhibit some similarities in their biochemical and assembly properties, our comparative LS studies have revealed striking differences in their dynamic behaviors. Disassembly of SeFtsZ commenced well before GTP depletion (Fig. 2, *A* and *C*), as observed for SyFtsZ (32). In this respect, cyanobacterial FtsZs behave similarly to EcFtsZ. Small and Addinall (23) found that increasing the GDP concentration in the presence of constant GTP resulted in earlier disassembly of EcFtsZ and concluded that the assembly state of EcFtsZ is sensitive to the GTP/GDP ratio and GDP accumulation. The resemblance of SeFtsZ and SyFtsZ disassembly dynamics to those of EcFtsZ suggests that cyanobacterial protofilaments are similarly sensitive to decreasing GTP/GDP ratio and increasing GDP as GTP is hydrolyzed, leading to their destabilization and disassembly. In contrast, AtFtsZ2 remained assembled well beyond GTP depletion (Fig. 3, *A* and *E*) (47), and its disassembly was not hastened by assembly in equimolar GTP/GDP compared with GTP alone (47), indicating it is much less sensitive to nucleotide status. Red algal GsFtsZA also remained assembled long beyond GTP depletion (45, 47) and could even form protofilaments at high GDP concentrations (45). Together, these findings suggest substantial differences in the regulation of cyanobacterial and chloroplast FtsZ assembly state by GTP hydrolysis and indicate that one of the two FtsZs in chloroplasts evolved to form protofilaments considerably more stable than those in their cyanobacterial predecessor.

The high stability of AtFtsZ2 and GsFtsZA in chloroplasts is counterbalanced by their coassembly with AtFtsZ1 and GsFtsZB, respectively (30, 31, 45, 47, 65). Unexpectedly, our coassembly experiments with SeFtsZ have begun to suggest that AtFtsZ1 and GsFtsZB enhance protofilament dynamics through distinct mechanisms. Mixed SeFtsZ–AtFsZ2 protofilaments at all ratios behaved similarly to SeFtsZ protofilaments in that they began disassembling before GTP was depleted (Fig. 3, *A*, *C*, and *E*). Intriguingly, GsFtsZB appears to have a similar effect on GsFtsZA. We calculated GTP depletion times in previously published assembly assays for the individual and coassembled GsFtsZs (45) and found that both GsFtsZB alone and GsFtsZA–GsFtsZB, like SeFtsZ and SeFtsZ–AtFtsZ2, began disassembling before GTP was depleted (Table S1). This suggests that GsFtsZB and SeFtsZ may both render mixed protofilaments more sensitive to GTP hydrolysis and GDP accumulation. In contrast, data in Porter *et al.* (47) show that AtFtsZ1 does not have this effect; predicted depletion times for AtFtsZ2–AtFtsZ1 at various ratios in 10 μM GTP were all less than 410 s, but in all cases, disassembly did not begin until 1300 s or later (Table S2). We also noticed that SeFsZ and AtFtsZ1 affected the rates of disassembly differently. SeFtsZ accelerated disassembly of SeFtsZ–AtFtsZ2, as indicated by the greater negative slopes of the LS traces with increasing SeFtsZ (Fig. 3*E*). GsFtsZB had a similar effect on disassembly of GsFtsZA–GsFtsZB (45). AtFtsZ1, however, had little to no effect on the rate of AtFtsZ2–AtFtsZ1 disassembly (47). Consistent with the latter result, when AtFtsZ1 was coassembled with SeFtsZ, it did not increase the rate of disassembly relative to that of SeFtsZ alone and in fact appears to have decreased it (Fig. 4*A*), even while it significantly increased SeFtsZ subunit turnover from mixed SeFtsZ1–AtFtsZ1 protofilaments assembled in *S. pombe* (Fig. 5, *F* and *G*). Collectively, these observations suggest that GsFtsZB and AtFtsZ1 promote chloroplast FtsZ dynamics in distinct ways. GsFtsZB may act by increasing the sensitivity of protofilaments to nucleotide status, as our results suggest for SeFtsZ, whereas AtFtsZ1 may act more directly, perhaps by weakening subunit interfaces (30). More work will be required to address these hypotheses. Whatever the mechanisms, both proteins have the net effect of enhancing protofilament turnover dynamics (30, 31, 65). Notably, while greater dynamics and turnover of bacterial FtsZs have been correlated with higher rates of GTP hydrolysis (29, 45, 52, 66, 67), our measurements of the GTPase activities of individual and coassembled protofilaments indicate that neither the GsFtsZB-like mechanism nor the AtFtsZ1-like mechanism for enhancing the dynamics of chloroplast FtsZs entails a significant alteration of GTP hydrolysis rate (Tables 1 and S1) (45, 47).

Our work has interesting evolutionary implications. The chloroplast FtsZ pairs very likely arose by duplication of a single *ftsZ* gene acquired from the cyanobacterial endosymbiont (44, 68, 69). But phylogenetic analysis indicates that this duplication occurred independently in the red and green lineages after they diverged more than a billion years ago (70) and led subsequently to the evolution of FtsZA and FtsZB in the red lineage and FtsZ2 and FtsZ1 in the green lineage (44). The fact that FtsZ2 and FtsZA both exhibit exceptional stability while FtsZ1 and FtsZB both promote turnover dynamics therefore suggests that the scaffolding and dynamics-promoting functions performed by the single ancestral gene became separated independently in both sets of duplicates. Our findings here, along with other data described previously, further suggest that the cyanobacterial-like dynamics–promoting function was preserved in red algal FtsZB, and that green-lineage FtsZ1 may have evolved a new way of promoting protofilament dynamics. Distinct FtsZ duplications are also evident in other FtsZ-based division systems, including those performing mitochondrial division in some algae and protists and cell division in archaea (44, 71, 72, 73), and it is possible that similar patterns of functional divergence arose as a result. Supporting this conjecture, the archaeon *Haloferax volcanii* was recently shown to require two FtsZs for normal cell division—one with a largely stabilizing role and one that propels cell constriction (74)—somewhat analogous to the chloroplast division FtsZs (30, 31, 65). Studies of FtsZ function in diverse organisms are likely to further uncover themes and variations among FtsZ-based division systems in cells and organelles.

## Experimental procedures

### Production and purification of recombinant FtsZ proteins

A DNA fragment comprising the *S. elongatus* PCC 7942 *FtsZ* coding sequence (synpcc7942_2378) preceded by a 6x His (*His*_*6*_) tag and flanked by sequences required for cloning (Table S3) was synthetically produced by gBlocks Gene Fragments Integrated DNA Technologies with codon optimization for expression in *E. coli* and cloned by Gibson assembly (75) into the pET11b (Agilent) vector. The resulting plasmid, pET11b-His_6_-SeFtsZ, was transformed into BL21 cells (Novagen) for expression of SeFtsZ in *E. coli*. Construction of the plasmids encoding *A. thaliana* AtFtsZ1 (At5g55280) and AtFtsZ2 (At2g36250) without their predicted transit peptides and with C-terminal His_6_ tags, and their cloning into pDB328 (18) is described in the study by Olson *et al.* (46).

The bacterial strain expressing SeFtsZ was grown overnight at 37 °C and subcultured into fresh LB. Cultures were grown until an absorbance reached ∼0.6 to 0.8 at 600 nm and were then cold-shocked for 10 min in an ice bath. Isopropyl-β-d-1-thiogalactopyranoside (GoldBio) was then added to a final concentration of 0.6 mM, and the culture was grown overnight at 30 °C. Cells were collected by centrifugation and resuspended in low salt buffer (LSB; 50 mM Tris [pH 7.5], 50 mM NaCl, 10% glycerol) and frozen at −80 °C. Expression of AtFtsZ1 and AtFtsZ2 was performed similarly, but cultures were grown for 36 to 42 h at 14 °C, as described in the study by Porter *et al.* (47).

Purification of all FtsZ proteins was conducted as detailed previously (47), dialyzed into LSB, and aliquots were stored at −80 °C. Prior to each use, purified proteins were centrifuged at 80,000*g* at 4 °C for 30 min to remove any precipitate, and protein concentrations were then measured using the bicinchoninic acid assay assay (Thermo Scientific) and implementing a 20% correction factor (46, 76). The concentrations of SeFtsZ and AtFtsZ2 ranged from 25 to 85 μM, and the concentration of AtFtZ1 ranged from 10 to 20 μM.

### GTPase measurement

A regenerative GTPase assay modified slightly from Ingermann and Nunnari (77) was utilized to determine GTPase activities of the FtsZs, as described in the study by Porter *et al.* (47). In brief, each 200 μl reaction contained the desired protein concentration adjusted with LSB to a starting volume of 137.4 μl in a well of a 96-well plate. Next, 42.6 μl of GTPase reaction buffer was added, resulting in a final reaction containing 1 mM phosphoenolpyruvate (Sigma; catalog no.: P7002), 0.4 mM NADH (Sigma; catalog no.: N8129), and 20 U/ml pyruvate kinase/lactate dehydrogenase (Sigma; catalog no.: P0294), in 50 mM Hepes–KOH, pH 7.5, 5 mM MgSO_4_, and 100 mM KCl. Finally, the reaction was initiated by the addition of 20 μl GTP (Sigma) in LSB to a final concentration of 50 or 500 μM GTP, and absorbance at 340 nm was measured (Molecular Devices, SpectraMax [M2]). The velocity of GTP hydrolysis (μmol GTP min^−1^) was determined (described in detail in the study by Porter *et al.* (47)) and plotted as a function of each individual FtsZ concentration, or the total FtsZ concentration of mixed reactions, and the slope of the linear range was taken as the GTPase activity (GTP min^−1^ FtsZ^−1^) for each set of reactions. The X-intercept was utilized to determine the Cc of FtsZ required for GTPase activity. Rates are shown as an average of multiple replications of assays performed on multiple protein preparations on various days (n = 2–7), demonstrating the consistency of the results. Errors are shown as SD.

### GTP depletion calculations

GTP depletion times were calculated based on total FtsZ concentrations above the average Cc, and average GTPase activities were determined for each reaction using the following equations:

FtsZ concentration above Cc (adjusted concentration)

$$\mathrm{Adjusted}\;concentration\;\left(\mu M\right)=Experimental\;FtsZ\;concentration\;\left(\mu M\right)-Cc\;\left(\mu M\right)$$

Predicted GTP depletion time

$$GTP\;depletion\;time\;\left(s\right)=\left(\frac{\left(\frac{Experimental\;GTP\;concentration\;\left(\mu M\right)}{\mathrm{Adjusted}\;concentration\;\left(\mu M\right)}\right)}{GTPase\;activity\;\left(\frac{GTP}{\frac{\min}{FtsZ}}\right)}\right)\times \left(\frac{60\;s}{\min}\right)$$

### Assembly buffer

Assembly experiments monitored by LS, sedimentation, and TEM were conducted in HMK buffer: 50 mM Hepes–KOH, pH 7.5, 5 mM MgSO_4_, and 100 mM KCl. All reactions were initiated by addition of nucleotide.

### Electron microscopy

Assembly reactions for TEM were carried out in assembly buffer in 20 to 100 μl total volumes, initiated by addition of nucleotide as described previously (47). Assemblies were performed at room temperature for the desired time, and 5 μl of the reaction was pipetted onto to a carbon-coated 400-mesh copper grid prepared in our laboratory and prepared for imaging as described previously (47). A JEOL 1400 Flash transmission electron microscope (Japan Electron Optics Laboratories) was used at magnifications from 6000 to 100,000× to visualize protofilaments, which were measured using ImageJ (NIH) (78). In an effort to control sample variability, a single assembly reaction was initiated with addition of GTP, and samples were taken at the indicated times for TEM images shown in Figure 2*E*.

### Light scattering

*In vitro* 90° LS assays were performed at room temperature as described previously (45, 46) with a fluorescence spectrophotometer (Photon Technology International) equipped with a model 814 photomultiplier utilizing the digital mode set at 1000 V. Instrument parameters, sample preparations, cuvette, and signal recording used were described previously (47). Note that the LS signal reflects both polymerization and bundling (32). All reactions were repeated at least twice on different days with different protein preparations, and similar results were obtained. Only data observed on the same day during the same experiment are plotted together. The initial rates of assembly were determined for each LS trace (minimum of two replicates), as described previously (47). The initial rates shown in each figure correspond to the LS assays presented in the same figure.

### Sedimentation assays

Prior to sedimentation assays, proteins were centrifuged at 80,000*g* for 30 min at 4 °C to remove any precipitates that may have formed during storage and subsequently quantified as described previously. The desired concentration of protein in LSB was first added to each reaction tube and allowed to warm to room temperature prior to initiation of each reaction. A one 10th volume of 10× HMK buffer was then added followed by addition of nucleotide at a 10× concentration, allowing for the desired final concentration. FtsZs and nucleotides were incubated at room temperature for 30 min and centrifuged at 80,000*g* in an S100AT4 607 rotor (Thermo Fisher Scientific) for 30 min at 4 °C. The supernatant was collected, and the pellet was then resuspended in the same volume of LSB. A sample of the protein not centrifuged was used as the total protein control. The total protein, supernatant, and pellet samples were analyzed by SDS-PAGE and quantified by densitometry using ImageJ software (78). Sedimentation assays were carried out three times, and the proportion of protein in the pellet fractions (density of pellet/density of pellet + density of supernatant) are reported. Errors are shown as SD. SeFtsZ and AtFtsZ2 have similar molecular masses and could not be resolved by SDS-PAGE in sedimentation assays.

### *S. pombe* expression constructs and FRAP assays

Vectors for expression of a single fluorescent protein were all made utilizing BamHI or BamHI and XhoI digestion of the expression vector pREP41X and subsequent Gibson assembly cloning (75, 79). The *S. elongatus* PCC 7942 FtsZ (synpcc7942_2378) fragment used for cloning into pREP41X was synthetically produced by gBlocks Gene Fragments Integrated DNA Technologies with codon optimization for expression in *A. thaliana* (Table S3). Primers used for construction of pREP41X-mV (KO3203) and pREP41X-SeFtsZ-mCerulean-SeFtsZ (KO3200) are shown in Table S4. pREP41X-SeFtsZ-mCerulean-SeFtsZ (KO3200) was constructed with the mCerulean tag located in an internal loop after amino acid 80 of SeFtsZ (80) with a GSGSGS linker on either end of mCerulean by initially cloning three fragments that were PCR amplified with the following primer pairs: LY165F and LY161R, LY65F and LY66R, and LY162F and LY166R (Table S4) followed by Gibson assembly (75). pREP41X-AtFtsZ1_FL_-mV was published previously (79) and is referred to here as AtFtsZ1-mV. The methodology described in the study by TerBush *et al.* (79) was utilized to construct a coexpression vector for analysis of two proteins within a single *S. pombe* cell. In brief, the entire expression cassette from pREP41X-SeFtsZ-mCerulean-SeFtsZ (KO3200) was amplified with primers AT109F and AT110R (Table S4) and inserted into either pREP41X-mV or AtFtsZ1_FL_-mV digested with AatII. The resulting vectors were as follows: pREP41X-mV+SeFtsZ-mCerulean-SeFtsZ (KO3201) and pREP41X-AtFtsZ1_FL_-mV+SeFtsZ-mCerulean-SeFtsZ (KO3202). Expression vectors were then transformed into *S. pombe* strain MYB192 (81) as described previously (79). Transformed *S. pombe* cells were mounted on l-lysine–coated coverslips (Sigma–Aldrich), and all imaging and FRAP experiments were performed at room temperature.

Images of protein expression in *S. pombe* were acquired using a FluoView 1000 laser-scanning confocal microscope (Olympus). All the images were captured under a 100× oil (numerical aperture: 1.42) objective with 1024 × 1024 pixels in FV1000 ASW software (Olympus). Further processing of the images to project the Z-stacks and merge fluorescence signals was performed in Fiji (https://fiji.sc) (82). For FRAP experiments, a FluoView 1000 laser-scanning confocal microscope (Olympus) and FV1000 ASW software (Olympus) were used. FRAP data were collected under a 100× oil (1.42 numerical aperture) objective with a 458 nm laser at 50% intensity for imaging of mCerulean-fusion proteins and with a 405 nm laser at 50% intensity for photobleaching. The photobleached areas were set as a circular spot 20 pixels (about 1.28 μm) in diameter with the Tornado scanning tool in the software. Prior to photobleaching, three images were taken at intervals of 10 s. The 20-pixel area was then photobleached for 20 ms, and 30 additional images were taken every 10 s. In order to correct and normalize the FRAP data, circular spots of the same size were chosen in a region with fluorescence signal but away from the bleaching site and in a background region free of fluorescence signal. The normalized FRAP data were analyzed in pro Fit 7 software (QuantumSoft). The best fitting curves were generated through the following equation for a two-binding state model, as described previously (31, 83, 84):

$$FRAP\;\left(t\right)=\left(1-r\right)\left(1-{∁}_{eq1}e^{{-k}_{off}t}-{∁}_{eq2}e^{{-k}_{off2}t}\right)$$

where *k*_*off1*_ and *k*_*off2*_ refer to dissociation rate constants, *C*_*eq1*_ and *C*_*eq2*_ refer to fractions of bound molecules, and *r* refers to an additional parameter for the effect of incomplete recovery. The resulting parameters are shown in Table S5.

### Statistical analysis

Statistical analyses for GTPase and assembly assays were performed using Prism GraphPad 7.0b software (GraphPad Software, Inc). Plots were also generated with Prism Graphpad software, where reported errors represent SD. All *p* values represent unpaired *t* tests. FRAP data were analyzed and plotted as described above.

## Data availability

Most data are contained within the figures and tables except where it is explicitly stated that representative data are shown. Raw data are available upon request from the corresponding author.

## Supporting information

This article contains supporting information (45, 47, 84).

## Conflict of interest

The authors declare that they have no conflicts of interest with the contents of this article.
