# Supplementary material for: Dynamics of the Synechococcus elongatus cytoskeletal GTPase FtsZ yields mechanistic and evolutionary insight into cyanobacterial and chloroplast FtsZs
Source: J Biol Chem. 2023 Jan 16;299(3):102917. doi: 10.1016/j.jbc.2023.102917 (PMC9975276; doi:10.1016/j.jbc.2023.102917)
Supplement: Supporting information [file mmc1.pdf]

Dynamics of the *Synechococcus elongatus* cytoskeletal GTPase FtsZ yields mechanistic and evolutionary insight into cyanobacterial and chloroplast FtsZs

Katie J. Porter[1,¶], Lingyan Cao[1,§], Katherine W. Osteryoung[1,*]

Supporting Information

Fig. S1. GTPase activity of SeFtsZ at 500 and 50 µM GTP.

Fig. S2. GTPase assays of SeFtsZ and AtFtsZ proteins mixed at different ratios.

Fig. S3. Sedimentation assays of SeFtsZ and AtFtsZ1 mixed at different ratios.

Fig. S4. FRAP images of SeFtsZ-mC in the absence and presence of AtFtsZ1-mV.

Table S1. Predicted times of GTP depletion and approximate times disassembly began in assembly reactions containing GsFtsZA, GsFtsZB or their mixtures calculated from data in Chen et al 2017 (45).

Table S2. Predicted times of GTP depletion and approximate time disassembly began in assembly reactions containing mixtures of AtFtsZ2 and AtFtsZ1 calculated from data in Porter et al 2021 (47).

Table S3. Sequences of synthetic SeFtsZ cloning fragments.

Table S4. Primers used for cloning.

Table S5. Resulting parameters of FRAP experiments.

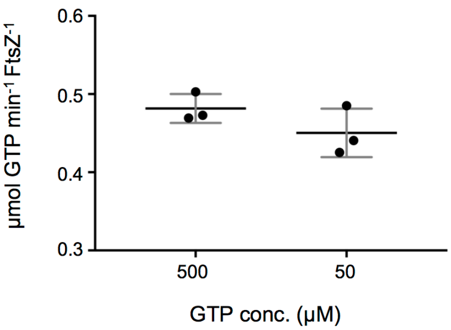

Fig. S1. GTPase activity of SeFtsZ at 500 and 50 µM GTP. Each of the three replicates at 500 and 50 µM GTP represents a different protein purification. There was no significant difference between activities at the two GTP concentrations as determined by an unpaired t test ($p = 0.520$).

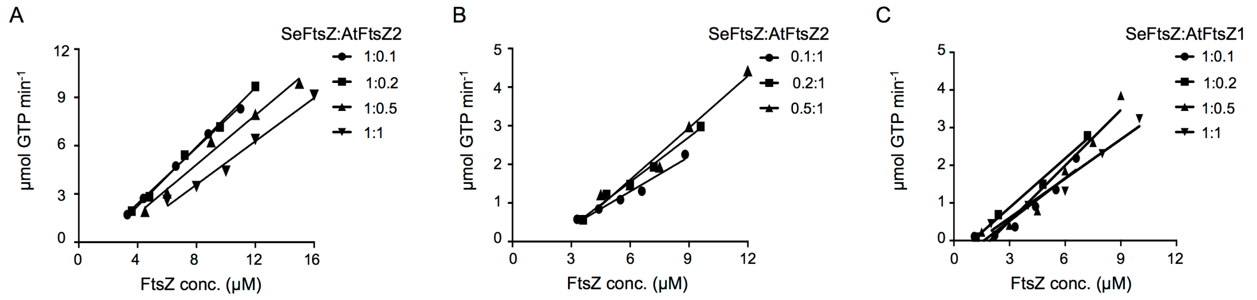

Fig. S2. GTPase assays of SeFtsZ and AtFtsZ proteins mixed at different ratios. Activities were assayed at the indicated ratios at 25° C in 500 µM GTP. The GTPase activity is the slope of the regression line above the Cc. Representative GTPase activities are shown for *A* and *B*, SeFtsZ mixed with AtFtsZ2, and *C*, SeFtsZ mixed with AtFtsZ1.

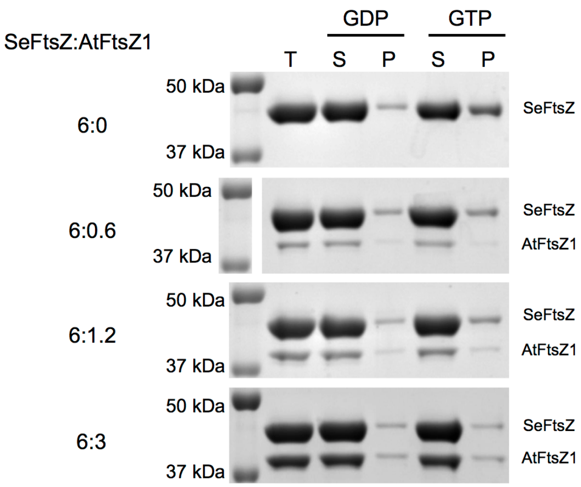

Fig. S3. Sedimentation assays of SeFtsZ and AtFtsZ1 mixed at different ratios. Reactions containing 6 µM SeFtsZ mixed with AtFtsZ1 at the indicated ratios were incubated for 30 min at room temperature after addition of either 500 µM GTP or GDP and centrifuged at 80,000 *g* for 30 min at 4° C. Proteins were then separated by SDS-PAGE. Representative Coomassie-stained gels show SeFtsZ and AtFtsZ1 proteins in the total (T), supernatant (S), and pellet (P) fractions. Markers (kDa) are shown on the *left*. The gel image in the top panel is repeated from Fig. 1*E* to show a complete representative set of assays performed on the same day with the same protein preparations.

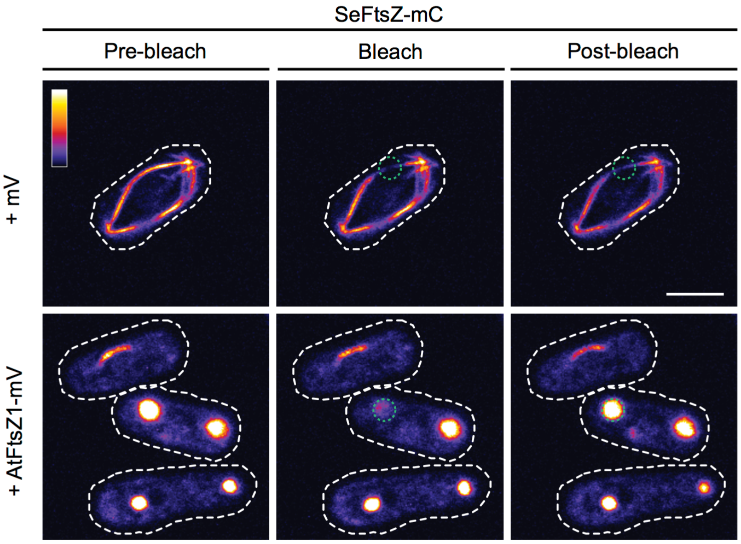

Fig. S4. FRAP images of SeFtsZ-mC in the absence and presence of AtFtsZ1-mV. Representative images of SeFtsZ-mC fluorescence in *S. pombe* cells coexpressing SeFtsZ-mC with mV (*top*) or AtFtsZ1-mV (*bottom*) are shown before photobleaching (pre-bleach), at the time of photobleaching (bleach) and 150 s after photobleaching (post-bleach). The green dashed circles outline the photobleached regions. The white dashed lines outline the cells in the imaged fields. The color scale bar (*top left*) indicates the SeFtsZ-mC fluorescence intensity from highest (white) to lowest (black). Bar = 5 µm.

Table S1. Predicted times of GTP depletion and approximate times disassembly began in assembly reactions containing GsFtsZA, GsFtsZB or their mixtures calculated from data in Chen et al 2017 (45). GTP depletion times were calculated based on total GsFtsZ concentrations above the average critical concentration (Cc) and average GTPase activities determined for each reaction. Reported GTPase activities and Cc were originally reported in Chen et al 2017 (45). Because reported GTPase activities were determined based on GsFtsZA concentration alone, for mixed reactions we calculated an "Adjusted GTPase Activity" by dividing "Reported GTPase Activity" by total GsFtsZ concentration. Approximate times disassembly began (Approx. Time Disass. Began) were estimated by visual assessment of when the light scattering signals began decreasing on traces in the indicated original figures.

	Reported GTPase Activity (GTP $FtsZ^{-1}$ min^{-1})	Adjusted GTPase Activity (GTP $FtsZ^{-1}$ min^{-1})	Total GsFtsZ (μM)	Cc (μM)	GsFtsZ above Cc (μM)	GTP (μM)	GTP Depletion Time (s)	Approx. Time Disass. Began (s)	Original Figure
GsFtsZA	0.69	na[1]	5	0.6	4.4	50	988	na[2]	*5A*, *5B*
GsFtsZA	0.69	na[1]	10	0.6	9.4	50	463	5300[3]	*3D*
GsFtsZB	0.41	na[1]	10	3.6	6.4	50	1143	180	*3E*, *5A*
GsFtsZA:GsFtsZB (5:2.5)	0.95	0.63	7.5	0.8	6.7	50	707	700	*5B*
GsFtsZA:GsFtsZB (5:5)	1.04	0.52	10	0.5	9.5	50	607	320	*5B*
GsFtsZA:GsFtsZB (5:10)	1.48	0.49	15	0.2	14.8	50	411	150	*5A*

[1]Adjustment not needed.
[2]Not observed during 2000 s assay.
[3]Assay monitored for 3 h.

Table S2. Predicted times of GTP depletion and approximate time disassembly began in assembly reactions containing mixtures of AtFtsZ2 and AtFtsZ1 calculated from data in Porter et al 2021 (47). GTP depletion times were calculated based on total AtFtsZ concentrations above the average critical concentration (Cc) and average GTPase activities determined for each reaction. GTPase activities and Cc were originally reported in Porter et al 2021 (47). Approximate times disassembly began (Approx. Time Disass. Began) were estimated by visual assessment of when the light scattering signals began decreasing on traces in the indicated original figures.

	GTPase Activity (GTP $FtsZ^{-1}$ min^{-1})	Total AtFtsZ (μM)	Cc (μM)	AtFtsZ above Cc (μM)	GTP (μM)	GTP Depletion Time (s)	Approx. Time Disass. Began (s)	Original Figure
AtFtsZ2	0.22	5	0.36	4.64	10	587.8	na[2]	3*A*, *green*
AtFtsZ2:AtFtsZ1 (5:0.5)	0.29	5.5	0.43	5.07	10	408.1	1420	3*A*, *orange*
AtFtsZ2:AtFtsZ1 (5:1)	0.26	6	0.19	5.81	10	397.2	1310	3*A*, *red*
AtFtsZ2:AtFtsZ1 (5:2.5)	0.27	7.5	0.043	7.457	10	298.0	1400	3*A*, *purple*
AtFtsZ2:AtFtsZ1 (5:5)	0.36	10	-[1]	10	10	166.7	1390	3*A*, *blue*

[1]The Cc determined in this reaction was close to 0, as reported in Porter et al 2021.
[2]Not observed during 2000 s assay.

Table S3. Sequences of synthetic SeFtsZ cloning fragments.

Sequence	Vector
**synpcc7942_2378 codon optimized for *E. coli*** (coding sequence underlined)	**pET11b-His$_6$-SeFtsZ**
ttaactttaagaaggagatatacatatgcatcaccatcaccatcacggcagtacggacccaatgccaatcaacaactcgtacgggtttaatcgcgatggttcactgtctggtt<br>ttgatgcgttgggacagcctgaagaactcattattcctagctcggttgcccgcatcaaagtgatcggtgtgggcggtggtggcagcaatggagtcaaccgtatgatttcaa<br>gcgatgtgtctggcgtcgaattttgggctttgaacacagatgcgcaagcactgttacattcggcggccccgaaacgtatgcagcttggtcagaaactgacccgtggtcttg<br>gagcaggcggtaatcccgctatcggcatgaaagcggcggaggaatcacgcgaggaattgattgcggcactggaaggagctgacttagttttcattacagccggaatgg<br>gcggtggtaccggtaccggcgctgcgcctatcgtcgcagaagtggccaaagaagtaggtgcgcttactgtggggattgtgaccaaaccgttcacatttgaaggccgcc<br>gtcgtatgaaacaggcggaagaaggcaccgcggcccttcagtcgtcagtggatactctgatcactattcccaacgaccgcttattacatgccatctctgaacagactccca<br>ttcaagaggcgtttcgtgtggcggacgatatcctccgtcaaggtgtacagggcatttcggatatcattaccatcccgggcctggtcaatgtggacttcgccgatgtccgcgc<br>ggtcatggccgatgccggcagcgcactgatgggcatcggttcagggtcgggtaagtcacgcgcgcgtgaagcagcacatgccgcgatctcaagcccgctgctggagt<br>ccagcatcgaaggtgcccgtggagtggtgttcaatatcacgggagggcgtgatatgacgcttcatgaagtcaacgctgctgccgacgccatctatgaagtggtagaccc<br>ggaagcaaatattatcttcggtgccgttatcgatgaccgcctcgagggagagctgcgcattaccgtaatcgcaaccggcttctcgactgatcgtccgaacctgaacacgat<br>cagcaccagtacgtcgcagccgaccagccagccaagcgtgtcgccaaacccagccagcgcacccccctgcgagcggtgggggtctggatatcccggcttttctgcagc<br>gcaaaattcagaaccgcccctaaggatccggctgctaacaaagcccga	
**synpcc7942_2378 codon optimized for *A. thaliana***	**KO3200**
atgactgatcctatgccaataaacaactcatacggttttaatagagacggttcattgagcgggtttgacgctctcggtcagcccgaagagttgattatcccctcttccgtcgca<br>agaattaaagtgattggagtgggaggagggggggtctaatggggtaaatcgtatgatctctagtgatgtgtctggagttgaattctgggcacttaatactgatgctcaagcttt<br>gcttcatagcgcagcaccaaagagaatgcagttggggcaaaaacttacccgaggcctgggcgctggaggaaacccagcgatcggaatgaaggcagcggaggaaag<br>tagggaagaactgattgcggctttagaaggagctgacctcgtttttattactgctggaatgggcggaggaacgggtaccggagctgctcctatcgtcgcggaagtagcaa<br>aagaagtaggagctctcaccgtgggaatagttacgaaaccttttaccttcgaagggagaagaaggatgaagcaggcggaagaagggaccgctgcactccaaagttctg<br>tggatacgctcatcaccatcccgaatgataggttgctgcacgcgatcagtgagcaaactccgatccaagaagcttttcgagtcgccgacgacatcctcagacaaggggtg<br>caagggatcagcgacattataacaattccaggcctagtcaatgtcgactttgccgacgtccgtgcagttatggcggacgcggggagcgccctaatgggaattggttctgg<br>ctctggcaagtcccgtgccagagaggcggctcacgcggccatttccagtcccctactagaatccagcatagaaggagcccgtggggtggtctttaacatcactggtggg<br>cgtgatatgacccttcacgaagtcaatgccgctgccgatgctatttatgaggtagttgatcccgaagcaaatattatcttcggagcggtaattgacgaccgactggagggc<br>gagctccgtataacagttatcgcaacgggttttagtacggatcgtcccaatctaaataccataagtacatctacttcccaaccaaccagccaaccttcagtctccccaaatcc<br>agcctctgctcccccagctagtgggggcggcttagacatccctgcctttcttcaacgaaaaatacaaaacagaccgtag	

Table S4. Primers used for cloning.

Primer ID	Sequence (5' to 3')	Vectors constructed
AT310 F	GTTAAATCATACCTCGAG*GGATCC*ACCATGGTGAGCAAGGGCGAGGAGCTG	KO3203
AT 297 R	GACATTCCTTTTACCCGG*GGATCC*TTACTTGTACAGCTCGTCCATGCCGAG	KO3203
LY165 F	GTTAAATCATACCTCGAGATGACTGATCCTATGCCAATAAACAACTCA	KO3200
LY 161 R	CTCGCCCTTGCTCACCATAGAACCAGAACCAGAACCTGCGCTATGAAGCAAAGC<br>TTGAG	KO3200
LY 65 F	ATGGTGAGCAAGGGCGAG	KO3200
LY 66 R	CTTGTACAGCTCGTCCATGCC	KO3200
LY 162	ATGGACGAGCTGTACAAGGGTTCTGGTTCTGGTTCTGCACCAAAGAGAATGCAG<br>TTGG	KO3200

Table S5. Resulting parameters of FRAP experiments. k_{off1} and k_{off2} refer to dissociation constants, C_{eq1} and C_{eq2} refer to fractions of bound molecules and r refers to an additional parameter for the effect of incomplete recovery (84).

Parameter	SeFtsZ-mCerulean (single expression)	SeFtsZ-mCerulean (coexpression with AtFtsZ1)
k_{off1}	0.4299	-0.0078922
k_{off2}	0.0063803	0.01501
C_{eq1}	0.079155	0.022846
C_{eq2}	0.8789	0.9435
r	0.828	0.1275
